# Systemic analysis of lipid metabolism from individuals to multi-organism systems†

**DOI:** 10.1039/d4mo00083h

**Published:** 2024-09-09

**Authors:** Samuel Furse, Carlos Martel, David F. Willer, Daniel Stabler, Denise S. Fernandez-Twinn, Jennifer Scott, Ryan Patterson-Cross, Adam J. Watkins, Samuel Virtue, Thomas A. K. Prescott, Ellen Baker, Jennifer Chennells, Antonio Vidal-Puig, Susan E. Ozanne, Geoffrey C. Kite, Milada Vítová, Davide Chiarugi, John Moncur, Albert Koulman, Geraldine A. Wright, Stuart G. Snowden, Philip C. Stevenson

**Affiliations:** a Royal Botanic Gardens Kew Kew Green Richmond Surrey TW9 3AE UK s.furse@kew.org samuel@samuelfurse.com p.stevenson@kew.org +44 (0) 20 8332 5867 +44 (0) 8332 5377; b Core Metabolomics and Lipidomics Laboratory, Wellcome-MRC Institute of Metabolic Science, University of Cambridge, Addenbrooke's Treatment Centre Keith Day Road Cambridge CB2 0QQ UK; c Department of Zoology, The David Attenborough Centre, University of Cambridge, Corn Exchange St. Cambridge CB2 3QZ UK; d Department of Zoology, University of Oxford Oxford OX1 3SZ UK; e School of Biological Sciences, Faculty of Environmental and Life Sciences, University of Southampton, University Road Southampton SO17 1BJ UK; f Wellcome-MRC Institute of Metabolic Science and Medical Research Council Metabolic Diseases Unit, University of Cambridge, Keith Day Road Cambridge CB2 0QQ UK; g Bioinformatics Core, Wellcome-MRC Institute of Metabolic Science, University of Cambridge, Addenbrooke's Treatment Centre, Keith Day Road Cambridge CB2 0QQ UK; h Lifespan and Population Health, School of Medicine, University of Nottingham Nottingham NG7 2UH UK; i Institute of Botany, Czech Academy of Sciences, Department of Phycology Dukelská 135 379 01 Třeboň Czech Republic; j Max Planck Institute for Human Cognitive and Brain Sciences Stephanstraße 1a 04103 Leipzig Sachsen Germany; k SpectralWorks Limited, The Heath Business and Technical Park Runcorn Cheshire WA7 4EB UK; l Department of Biological Sciences, Royal Holloway College, University of London Egham Surrey TW20 0EX UK; m Natural Resources Institute, University of Greenwich Chatham Kent ME4 4TB UK

## Abstract

Lipid metabolism is recognised as being central to growth, disease and health. Lipids, therefore, have an important place in current research on globally significant topics such as food security and biodiversity loss. However, answering questions in these important fields of research requires not only identification and measurement of lipids in a wider variety of sample types than ever before, but also hypothesis-driven analysis of the resulting ‘big data’. We present a novel pipeline that can collect data from a wide range of biological sample types, taking 1 000 000 lipid measurements per 384 well plate, and analyse the data systemically. We provide evidence of the power of the tool through proof-of-principle studies using edible fish (mackerel, bream, seabass) and colonies of *Bombus terrestris*. Bee colonies were found to be more like mini-ecosystems and there was evidence for considerable changes in lipid metabolism in bees through key developmental stages. This is the first report of either high throughput LCMS lipidomics or systemic analysis in individuals, colonies and ecosystems. This novel approach provides new opportunities to analyse metabolic systems at different scales at a level of detail not previously feasible, to answer research questions about societally important topics.

## Abstract 2

Complete pipeline for system-level of biological systems, from indivituals to ecosystems, using only metabolite data.

## Graphical abstract

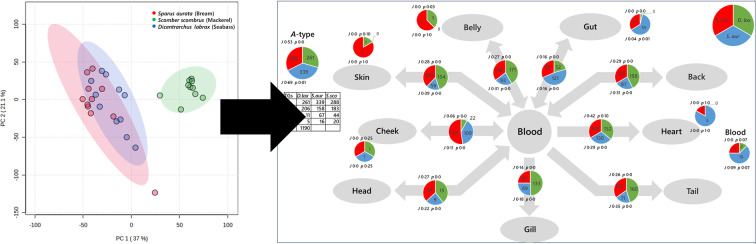

## 1. Introduction

Investigation of metabolic systems is a key part of studies into several globally important societal questions. For example, biodiversity loss is more acute than ever, increasing the urgency of studies on its underlying mechanisms. Studies on biodiversity loss involve investigating ecosystems, in which nutrients are passed between organisms. A closely related and important topic is global food security, which requires sustainable food production, including rearing of both livestock and crops. Sustainable food production requires a detailed understanding of health and metabolism within individual organisms as well as their environment and the interaction between the two. A common theme among investigations of biodiversity loss and global food security is the need for systemic analyses within or between individuals. Typically, investigating biological systems includes tackling questions about how those systems behave when they are challenged and how they are controlled in response to both intrinsic and extrinsic factors.

There has been an exponential expansion of genetics techniques and tools available for investigating how systems are controlled. These have been used in a wide range of applications, including to improve the production of foods^1–3^ and to investigate climate change^4–6^ and have given an invaluable insight into those systems and how they are constructed. However, genetics approaches are not able to directly measure how that system will respond to environmental challenges such as an increase in temperature. This requires more direct readouts of modifiable factors such as metabolites, *i.e.* the abundance and distribution of individual small molecules. Such an approach will provide mechanistic insight into the phenotypic effect(s) observed. Recently, investigations of how lipid metabolism is controlled have been reported.^7–9^ These studies used systemic or network analyses to answer questions about how metabolism is controlled and challenged in the context of dietary challenges, either through general changes (*e.g.*, a high fat diet) or individual nutrients (*e.g.*, individual poly-unsaturated fatty acids).

To answer questions about how metabolism is controlled or challenged in individual organisms or ecosystems, analysis of metabolites such as lipids is required from a range of sample types. This requires automation to make the scale of analyses feasible and subsequent wide-scale analysis *in silico* possible. Lipids are a key focus in biology because they include molecules used to supply and store energy (triglycerides), and others with a structural role (*e.g.*, phospholipids). Furthermore, as all cells need energy and membranes, studies on lipid metabolism are important for all cells. The study of lipid metabolism therefore provides a broad and detailed way to investigate the health and behaviour in biological systems from individual organisms to whole ecosystems, *i.e.*, across a range of scales.

Investigating lipid metabolism in ecosystems and individual organisms requires sample preparation techniques that cover the full range of sample types found in nature. This is a relatively new challenge and represents an emerging need for technological advancement as most lipidomics pipelines are designed for human blood serum and so have not been optimised for a range of sample types required for complex biological systems. Some ground work has been done on extending the range of tissue types in lipidomics studies,^10,11^ however none of these encompass diverse sample types such as plant material and insects.

A second challenge that emerges from the need to investigate whole ecosystems is the need to collect data from large numbers of samples in parallel. For example, high throughput techniques have emerged recently in metabolomics, with several studies using thousands of samples.^12–15^ For these analyses, extractions need to be automated^16^ with the minimum of steps to prepare samples.^17^ These and other methods have been reviewed^18–20^ and even tested.^11,21,22^ Direct Infusion Mass Spectrometry (DIMS) and semi-quantitative LCMS approaches have been reported for collecting lipidomics data. DIMS is an excellent tool for collecting lipidomics data from large numbers of samples without chromatography, and has been used in several of the largest lipidomics studies done to date.^13,14^ DIMS is a sensitive method that trades number of variables measured for the speed of data collection. Semi-quantitative high throughput LCMS has also been reported,^23^ measuring a greater number of lipids than DIMS, but requiring longer acquisition times per sample and with lower sensitivity.

For systemic analyses, a comprehensive survey of lipids is required, along with efficient and effective identification. Big and urgent societal questions on climate change and global food security require scope for network analysis as well as candidate biomarker analysis and similar statistical tests. This points to the need for measurement of as many lipids as possible in the system, and as consistently as possible.

**Fig. 1:**
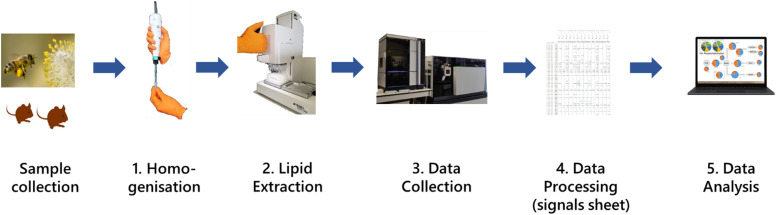
The pipeline for high throughput data collection of LCMS data from large numbers of biological samples. Samples collected from the field are stored at −80 °C (freeze-dried if needed), then (1) homogenised, (2) the lipids extracted, (3) profiled using LCMS, (4) the data extracted and processed to give a signals sheet with metadata, and then (5) analysed.

To meet the needs of systemic analysis of ecosystems and individual organisms, we suggest that three major advancements are required to construct a lipidomics pipeline suitable for the task. First, the best extraction method for collecting the lipidome for high throughput LCMS in a 384 well plate format must be determined. Second, a rapid and reliable way to process raw lipidomics data to give a signals sheet with all lipid variables ID-matched. Third, a way to undertake network analysis *in silico* on the data acquired. We have responded to these needs by constructing a pipeline for metabolomics-based analysis of both individual organisms and multi-organism systems (Fig. 1) and using it for proof-of-principle studies on big questions in ecosystem performance and the health of individual organisms.

We successfully applied our approach as proof-of-concept studies that highlight how lipid-based systems biology can be applied to address specific questions and hypotheses in biodiversity loss and other societally important questions.

## 2. Results and discussion

The construction of the lipidomics pipeline is described sequentially, starting with sample preparation with the selection of lipid extraction method, followed by data processing. Acquisition of lipidomics data from a range of sample types that describe both laboratory and ecosystem studies is then explained (Table S1, ESI†). How lipidomics data can be used to answer timely and important questions about lipidomics is then shown through two example proof-of-principle studies.

### 2.1. High throughput lipid extraction and data processing

We investigated methods for lipid extraction to identify the one most suitable for high throughput lipidomics using 384w plates. This was done in tandem with development of data processing in order that the latter served the former. Three solvent systems established for extracting lipidomes were tested, along with a more environmentally sustainable alternative that is not currently in widespread use (ethyl acetate, EAT). These solvent systems were the Bligh and Dyer^24^ (BAD), *tert*-butylmethyl ether^16^ (TBM), dichloromethane-methanol-triethylammonium chloride^10,25^ (3 : 1 : 0.002, DMT). These four extraction methods were tested on nine different sample types (mouse brain, heart and liver, cows’ milk, whole *Desmodesmus quadricauda*, leaves from *Eucalyptus perriniana*, polyfloral pollen, whole *Bombus terrestris*, whole *Saccharomyces cerevisae*; BRA, HEA, LIV, BTM, DQu, EuL, PFH, WHB, YEA respectively), with ten measurements of each stock. Extracts from all extraction methods were run on the same 384w plate. The extraction performance measures used were (i) the number of variables found, (ii) the total signal and (iii) the coefficient of variation, *i.e.*, a measure of how consistent the methods were. The data were then processed using two processing methods before numerical analysis and determination of which extraction method performed best.

Data from the extraction methods was initially processed using a conventional processing method.^26^ The number of signals (with a unique *m*/*z* and *R*_t_, Fig. S1A, ESI†) showed little difference between methods, unlike the total signal which did differ between methods (Fig. S1B, ESI†). Coefficients of variation (CV) of signal size were calculated for each variable in each method on each sample type (Table S2, ESI†). These showed that the BAD and DMT methods were similar, with slightly more variables having a CV below 20% and 15% for the DMT method. This type of analysis provided some insight into the difference between methods, however this approach to processing LCMS data is incompatible with a systems analysis as the latter requires ID-matching for all variables and this approach identified secondary ions for more abundant signals. To overcome this limit, we automated the matching of lipid IDs to lipidomics data using commercially-available software (AnalyzerPro® XD from SpectralWorks Ltd) with a comprehensive target library (TL) generated in-house. The TL consisted of around 7.5k triglycerides, ceramides and phospholipids and was used to assess extraction methods.

ID-matched processed data were then used to assess the quality of the extraction procedures. Fig. S2 (ESI†) shows the number of variables and total signal of ID-matched signals for each method. These analyses show subtle differences between the total signal measured for each of the methods, with BAD and DMT being similar and DMT often but not always slightly higher than BAD. Student's *t*-tests showed that DMT gave greater total signal for BRA, BTM, DQU, EuL, HEA and WHB (*p* 0.015272, 0.001395, 2.63 × 10^−6^, 3.53 × 10^−13^, 4.94 × 10^−6^, 2.16 × 10^−5^, respectively) whereas BAD gave greater total signal for YEA (*p* 0.001856). No difference in total signal was found between DMT and BAD for either LIV or PFH (*p* 0.352035, 0.684561). The total signal strength of extracts collected using EAT was higher than those of the TBM method, but not as high as BAD or DMT.

Processing the data using a TL simplified and reduced the computing power needed to produce a signals sheet. This facilitated assessment of the consistency of the extraction procedures (CV). The CV of the four methods calculated using only lipid variables, shows that the BAD and DMT methods performed similarly, with DMT giving 1–3% more lipid variables overall than the BAD (Table S3, ‘Sum’, ESI†). Here too, the EAT method was more consistent than the other three methods, and TBM was less consistent. The impressively consistent performance of the EAT method is encouraging, however the total signal being less than for other methods suggested that this solvent was saturated. So, of the methods tested, the DMT method performed best and was thus the one used. These results answer the question of which of the extraction methods tested is the best for data collection of high throughput LCMS lipidomics collection across a range of sample types needed for analysis metabolic systems.

### 2.2. Data analysis

**Fig. 2:**
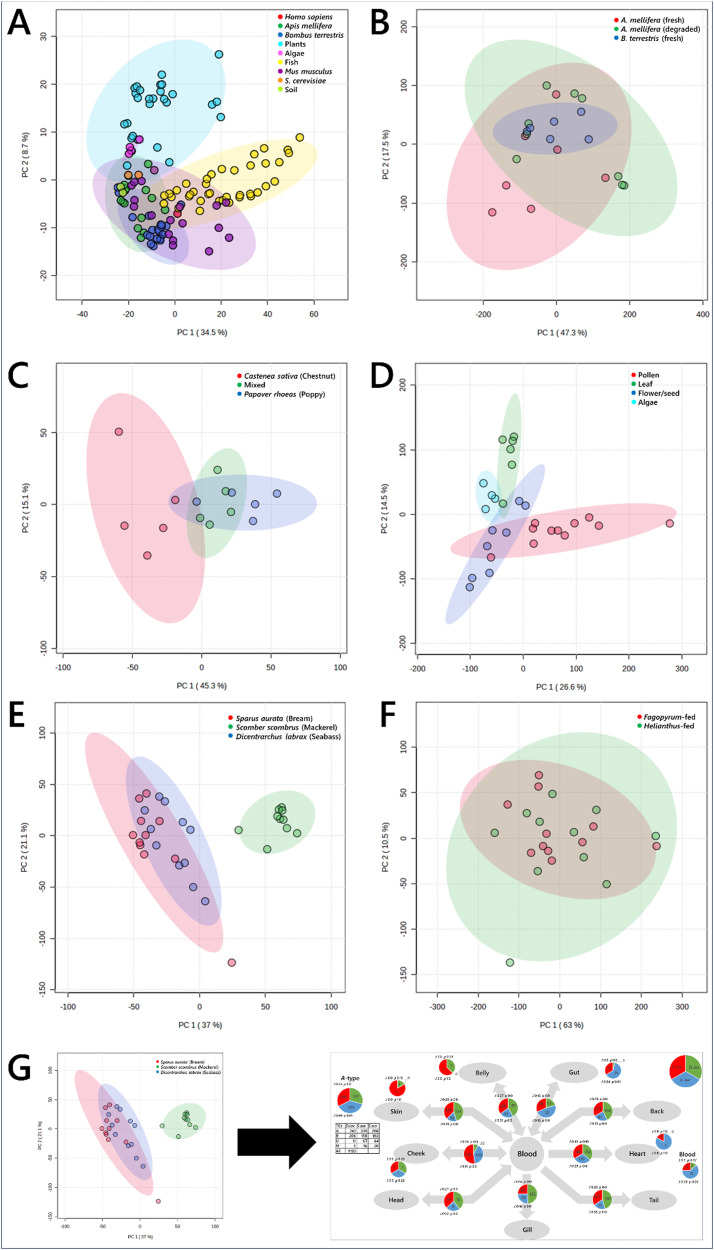
Principal component analyses of biological samples, drawn from plants, fish, mammals, yeast, bacteria and insects. Panel (A), samples of the nine different groups; (B), whole *Bombus terrestris*, fed one of three pollen diets; (C), plant and algal tissues; (D), *Apis mellifera* and *Bombus terrestris* tissues; (E), tissues samples from edible fish; (F), tissue samples from queen bees (*Bombus terrestris*) fed either mono-floral pollen from either *Fagopyrum tataricum* (buckwheat) or *Helianthus annuus* (sunflower) plants; (G), schematic representation of the exploitation of the known connections between tissues to undertake a traffic analysis. 95% confidence intervals are shown with ellipses of the same hue as the associated sample points. Data were log_10_-transformed (panel (A)) or signal corrected (panels B–F).

The depth and breadth of lipidomics data collection made possible by this pipeline allowed us to determine the lipid composition of a variety of sample types from different phylla, including plants, algae, fish, mammals and insects (Table S1, ESI†). Typically, analyses of data of this sort involves statistical tests, usually starting with a multi-variate analyses such as a principal component analysis (PCA). This type of test reduces dimensionality and can be used to identify sub-groups of samples and also to identify which variables drive the difference between two or more groups. Fig. 2A is a PCA of all samples run, showing insects, plants, algae, mouse, fish and even a human sample. These samples describe the range of sample types observed in studies of model laboratory organisms (mice) as well as of ecosystems. The PCA showed that the lipidome differed between these organisms. Plants overlapped entirely with algae but very little with animals of any kind. There was some similarity between the tissues of mice, bees, humans and fish, but as expected, they are generally distinct. PCAs also showed subgrouping within this, including between species of social bee (*Bombus terrestris* and *Apis mellifera*) and between storage conditions (Fig. 2B), feeding of *Bombus terrestris* (Fig. 2C), and plant tissues and algae (Fig. 2D). This type of analysis therefore provides a way to distinguish samples by identifying the lipids that differ the most between them. For example, this shows clearly that the dietary intake of lab-reared social bees was associated with contrasting lipid compositions *in vivo* (Fig. 2B).

However, multi-variate analyses such as PCAs give very limited insight into the mechanism that drives the effect seen. This provides a problem for system-level studies. Interpreting lipidomics data from several different tissues within individual organisms using an MVA is limited in what it can explain about how the system is controlled, as any visible distinction relies on subgrouping of individual tissues in the different groups. Similarly, ecology studies of landscapes that comprise several trophic levels requires a strong distinction between the molecular comparison of individual samples in order to see any difference between them. This type of analysis may therefore miss a range of sub-lethal differences between groups or locations ascribed to differences in dietary intake or nutrient availability form the landscape. For example, an important question in ecology at present is how pollination services are responding to climate change and how they can be maintained in order to protect the biodiversity of flowering plants. Thus the behaviour of both social and solitary bees with the rest of their environment and whether they visit a range of plants (generalist) or are more restricted (oligolectic), by preference or necessity, demands a more systemic approach than multi-variate analyses can give.

Second, MVAs fail to exploit the relationships between the samples, *i.e.*, the structure of the biological system from which they come. Fig. 2E and F show tissues that describe the metabolic structure of edible fish and *Bombus terrestris* fed contrasting diets, respectively. The difference between the groups can be seen, however what is accumulated where and thus how the system is controlled is not visible.

In order to understand how biological systems are controlled and what happens when they are stressed, the known connections between tissues or organisms must be exploited. Including the spatial distribution in the analysis sorts the metabolite composition data and allows it to be plotted such that the parts of the system when the biggest changes are found can be identified (shown schematically in Fig. 2G). We also judged that an approach that does not rely on controversial features such as *p* values associated with Students’ *t*-tests is also attractive. We therefore updated and expanded a non-statistical approach to network analysis for analysing metabolic systems, and present Lipid Traffic Analysis v3.0 (LTA). This software plots the spatial distribution of variables according to their lipid type. ***A***-type variables are lipids found in all compartments (tissues/sample types) of a given group. ***B***-type lipids are variables found in pairs of adjacent compartments, for example in the liver and the serum in mammals or the brain and ocular cortex in bees. ***U***-type variables are found only in one compartment for a given group. We also introduce ***N***_**2**_-type variables that are for variables found in pairs of non-adjacent groups. The ***N***_**2**_-type is useful for identifying variables that exist independently or imply the existence of unexpected connections in a network.

Analysing lipid data in this way is useful because (i) it is a plot of lipid distribution that does not rely on probability or other metrics, (ii) the plots can be used to characterise the system and (iii) the analysis sifts out the most important variables and parts of the network, identifying how the control of the systems differ. This approach therefore avoids a reliance on probability and so the need for significance thresholds is avoided. The combination of the data collection strategy we have developed and the network analysis, *i.e.*, the full pipeline, was used for two sets of proof-of-principle experiments for globally important societal challenges. One was on rearing livestock (fish) and the other on protecting biodiversity through understanding a generalist pollinator (bumble bee). These are two separate questions that require a similar approach and that this pipeline can be used to answer.

**Fig. 3:**
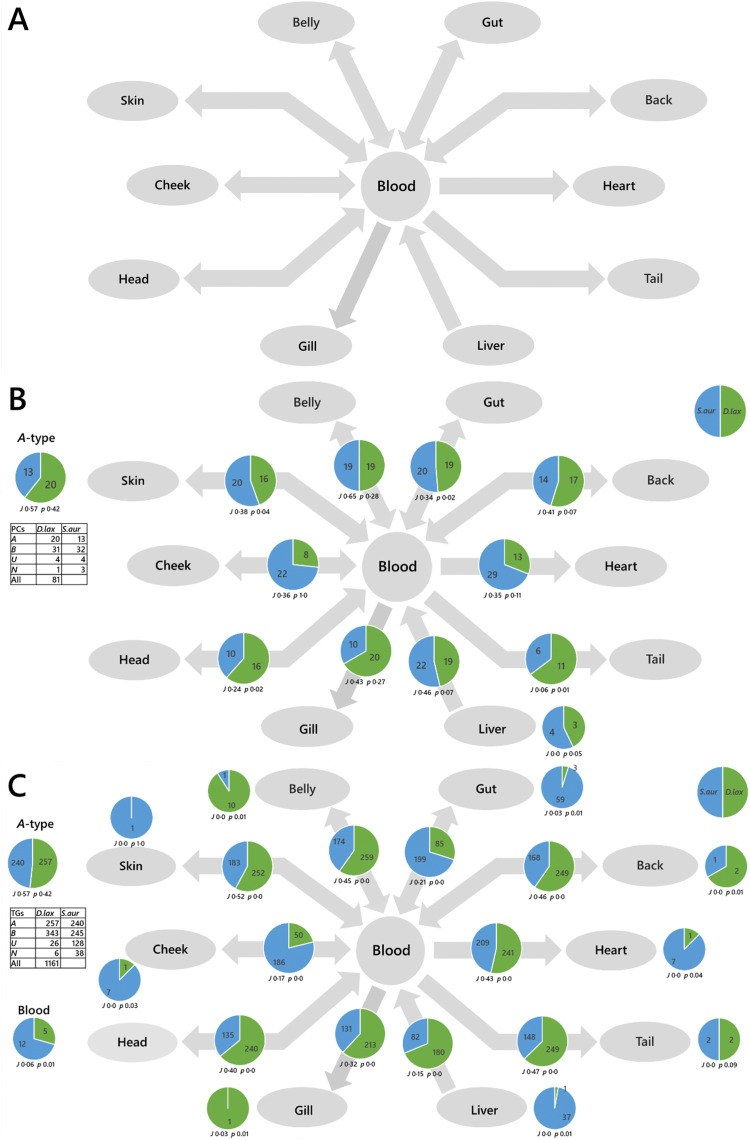
Switch Analyses (SA) of lipid pathways in *Dicentrarchus labrax* (seabass, *D. lax*) and *Sparus aurata* (bream, *S. aur.*). Panel (A), biological network; (B), switch analysis of phosphatidylcholine; (C), switch analysis of triglycerides. The pie chart in the top left shows the number of ubiquitous lipid variables for that network, for each phenotype (***A***-type variables). Pie charts on arrows represent variables found in the two adjacent compartments (***B***-type variables). Smaller pie charts represent isolated variables (***U***-type). *J* represents the Jaccard–Tanimoto coefficient for the comparison, with accompanying *p* value, as a measure of the similarity between the variables identified in the two phenotypes for each comparison. The *p* value shown represents the probability that the difference between the lists of variables for the two phenotypes occurred by random chance. TGs include all adducts of whole TGs and the DGs arising from in-source fragmentation of TGs during data collection.

First, a proof-of-principle traffic analysis on edible fish species from the same biome but different taxonomic orders (moroniforme and perciforme) was performed, and then with an Atlantic species of another order (scombiforme). The LTA of *Dicentrarchus labrax* (seabass) against *Sparus aurata* (bream) showed that there is a surprising uniformity of the PCs found throughout the system in both species, with several phosphatidylcholines (PCs) found throughout the system in both species (***A***-type lipids, Fig. 3A). However, there is no general pattern of PCs throughout the network between *D. labrax* and *S. aurata*, and only a modest overlap (*J*) between the two species. This suggests that lipid metabolism has evolved differently in the two taxonomic orders. Importantly, the traffic analysis of triglycerides between *D. labrax* and *S. aurata* also showed that there are over 200 triglycerides found throughout each species (Fig. 3B), something that is also observed in *S. scombrus* (mackerel, Fig. S3, ESI†).

These analyses show that there is a remarkable complexity in the lipid metabolism of edible fish in general and hints that for these fish to be healthy, the fatty acid profile of their dietary intake may also need to be very rich. This type of analysis therefore offers ways to manage the transition to eliminating the use of wild fish in farmed fish feeds without negatively affecting farmed fish growth or nutritional profile.^27^ Determining the precise dietary intake even of humans is notoriously difficult^28^ and thus that of a wild or farmed animal is yet more challenging. Gaining a greater understanding of the specific lipid requirements for farmed fish for optimum growth is critical for the aquaculture industry as it moves towards reducing its economically and environmentally costly reliance upon fishmeal and fish oil.^29,30^

Systemic analysis of a colony or mini-ecosystem of individuals is useful in studies related to biodiversity loss as it can tell us about the relationships between individuals. For example, pollinating insects such as bees provide an important service to plant-based habitats that are themselves a system. However, the living arrangements of bees also has a well-defined structure that represents a system. There is also scope for analysis of individual organisms. A proof-of-principle study in a commercially available species of bumble bee (*Bombus terrestris*) was done both within the queens and the whole colonies of which they were part. The colonies (*n* = 1 per group) were fed honeybee-collected pollen from *Fagopyrum esculentum* (buckwheat) or *Helianthus annuus* (sunflower).

The traffic analysis of lipids within the queens showed that for triglycerides, a diet of pollen from *Fagopyrum esculentum* was associated with a greater number of triglycerides throughout the system (Fig. S4A, ESI†). However, the traffic analysis of phosphatidylcholine suggested a more mixed picture for that lipid class (Fig. S4B, ESI†) and those of both phosphatidylinositol and phosphatidylglycerol (Fig. S4C and D, ESI†) suggest that the distribution of these lipids is more complicated than simply more or fewer variables. These analyses suggest that the control of lipid metabolism changes according to dietary intake and that this differs between triglycerides (energy storage and distribution) and phospholipids (cellular structure). This has potentially far-reaching consequences as it means that feeding in bees may have short- and long-term consequences on the individual bees. This raises questions about whether the effects are similar at colony level for social insects.

**Fig. 4:**
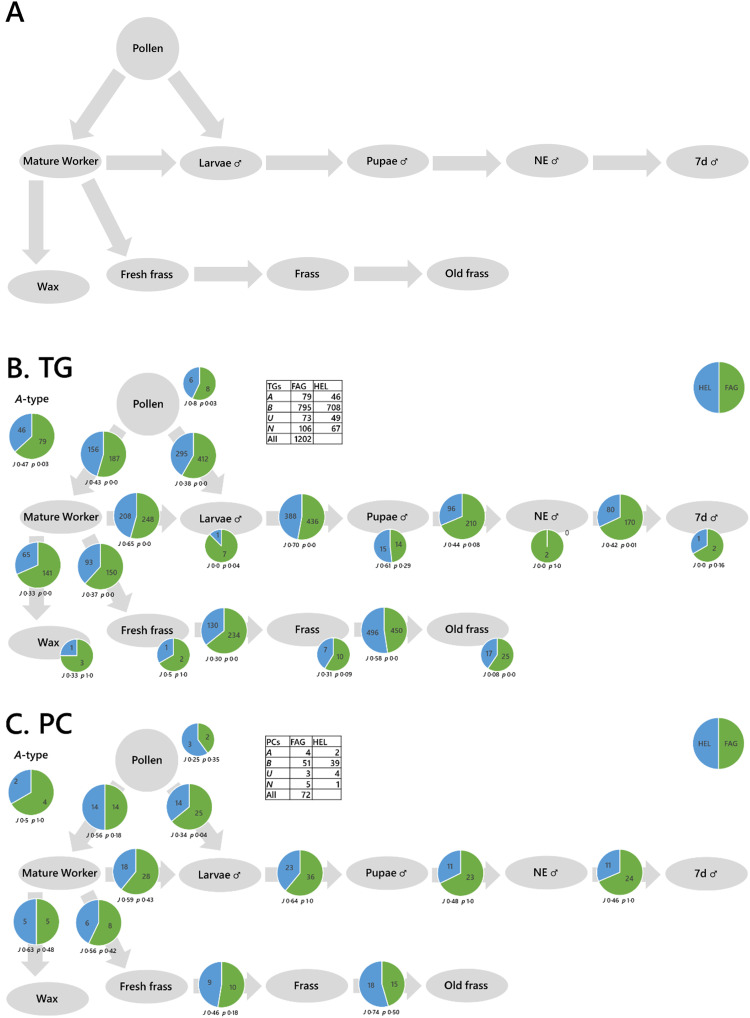
Switch analyses of phospholipid and triglyceride variables in *Bombus terrestris* colonies fed either *Fagopyrum tataricum* (FAG) or *Helianthus annuus* (HEL) pollen. Panel (A), Biological network; (B), switch analysis of triglycerides; (C), switch analysis of phosphatidylcholines. The pie chart in the top left shows the number of ubiquitous lipid variables for that network, for each phenotype (***A***-type variables). Pie charts on the arrows represent variables found in the two adjacent compartments (***B***-type variables). Smaller pie charts represent isolated variables (***U***-type). *J* represents the Jaccard–Tanimoto coefficient for the comparison, with accompanying *p* value, as a measure of the similarity between the variables identified in the two phenotypes for each comparison. The *p* value shown represents the probability that the difference between the lists of variables for the two phenotypes occurred by random chance.

Traffic analysis showed a simpler picture for the colony than within the queens (Fig. 4), with a greater number of variables throughout for TG and PC in the colony fed pollen from *Fagopyrum esculentum* than that fed pollen from *Helianthus annuus*. This is reflected in the traffic analyses of PG and PI (Fig. S5, ESI†). This therefore also shows that there are considerable diet-driven effects on the control of metabolism at colony level. These bee colonies also showed at least two fundamental features. First, both the phosphatidylcholine and triglyceride traffic showed that lipid composition of pupae, newly-emerged drones and week-old drones were similar, however the lipid composition of larvae was rather different to that of pupae whichever diet was fed. This suggested that there are considerable changes in lipid metabolism late in the larval development of bumble bees. Second, we see many more variables in 1d old frass and 7d old frass than in fresh frass. This suggests that new lipids are being made in the frass after it is produced. As several new phosphatidylcholines are found, we suggest that a eukaryotic species is probably responsible for this change in lipid composition, presumably a fungus. Bumble bee colonies may therefore represent a micro-ecosystem rather than simply a colony of one organism. Together with other evidence,^31^ this suggests that fungi play an important role in colony development of bumble bees.

Taken together, the evidence that dietary intake influences the control of lipid metabolism in colonies and individuals contextualises concerns about global challenges such as agricultural intensification and climate-change that can dramatically influence the nutrient landscape for bees. It suggests that changes to nutrient availability caused by biodiversity loss will have effects on the health of colonies of generalist pollinator bee species. This indicates that supporting pollination services is a key component of halting biodiversity loss.

The systemic analysis of both individuals such as fish, bees and an ecosystem has myriad applications for several timely questions in addition to understanding biodiversity loss and global food security. Lipid traffic analysis has already been used in medical research, on type 2 diabetes^32^ and gestational diabetes^7,33^ and feeding of essential nutrients.^9^ Studies of obesity and associated factors also require analysis of whole organisms and thus will rely on network analyses. Similarly, conditions such as cancer and infectious disease are system-wide and thus understanding of these diseases using systemic analyses can be part of an hypothesis-driven investigation of the progress of the disease and interventions to halt it. To date, much of the work on obesity, cancer, metabolic disease and infection has focused on lipid signatures of the conditions^34–36^ or on genetics.^37–39^

## 3. Conclusion

This study establishes a lipidomics pipeline that can measure the concentration of thousands of lipids in large numbers of samples, 1 000 000 per 384w plate, and then perform network analyses on the processed data to answer scientific questions. This novel approach represents a substantial advance in our ability to carry out the systemic metabolic analysis of individual organisms, colonies and even ecosystems. Thorough and objective testing of lipid extraction methods was used to identify the best method for resolution and consistency. The advances described relied upon the development of end-to-end methods for sample preparation and lipidomics data collection of a wide variety of tissue types—everything from leaf to liver—promptly and precisely. This enabled new insights in the proof-of-principle studies done that show that triglyceride metabolism was more varied and complicated in edible fish than expected, and that colonies of bees represented mini-ecosystems rather than simply groups of co-habiting individuals. The study of bee colonies also found that there is considerable development of lipid metabolism through the development of the bees. The advances in breadth and capacity in lipidomics that this pipeline offers provides the necessary infrastructure to answer key questions about how metabolic systems are controlled and what happens when they are challenged. This technology has immediate application in research into metabolic disease, nutrition, conservation, sustainable farming and biodiversity loss, amongst others.

## 4. Experimental

We report a pipeline for the systemic analysis of ecosystems and individuals using metabolomics. It consists of five steps (i) sample preparation, (ii) metabolite/lipid extraction, (iii) data collection, (iv) processing and (v) data analysis (Fig. 1). The advances that represent the development of unique steps—for which there are currently no similar approaches—are reported in methods. Analogous methods for extracting lipids from biological samples exist, as do different ways to process metabolomics data. We therefore investigated which was best and report those tests in Results. Proof-of-principle studies, in which the pipeline is used to investigate current questions, are also reported in Results.

### 4.1. Sample preparation

We sought a method that could be applied across a wide range of sample types, makes the lipid fraction chemically accessible and produces a pipettable solution and preserves the lipid fraction of the sample. We based our approach on a prototype developed for mammalian tissues in which the sample was dispersed in a buffer.^10,40^ This approach involved homogenising the samples in an aqueous medium comprising guanidine and thiourea, known as GCTU. This buffer is valuable because it suppresses lipase activity and bacterial growth, dismantles cellular structures at a molecular level without damaging lipids and supports preparation of a pipettable solution. However, adaptation of the existing method to cover the format of all the sample types that describe an ecosystem was required.

Leaf material and insect samples have not previously been used in large-scale lipidomics studies and presented unique challenges. Leaves and whole bees were made more brittle and partly preserved by being freeze-dried. Leaves were sliced to shorten the fibres (<5 mm) or crushed when dry, before being soaked in the buffer (2–6 h). The [dry] samples were then homogenised using a robust laboratory homogeniser (steel macerator). Bees required some blunt mechanical disruption immediately before mechanical homogenisation to break the head casing, and thoracic and abdominal exoskeleton. The constituent tissues of bees (brain, gut, hypopharyngeal gland, thoracic muscle, frass) and earlier developmental stages (larvae, pupae, newly emerged adults) behaved similarly to mammalian tissues (*Mus musculus*; brain, liver, adipose, heart, *Homo sapiens*; whole blood). Fish tissues (from *Dicentrarchus labrax*, *Scomber scombrus* and *Sparus aurata*; belly, gut, back, heart, tail, gill, head, cheek, skin, liver) also behaved in the same way. The amount of buffer used varied according to the amount of lipid in the sample, with fattier/more lipidic samples needing to be more dilute (see Table S1, ESI†).

### 4.2. Lipid extraction and data collection

A small number of lipid extraction methods have been reported for parallel or high throughput lipidomics application. However, although some objective tests of the performance of these methods have been done for medium throughput applications, and within other studies,^11,21,22^ no thorough performance review of lipid extraction has been done for large, high throughput studies or pipelines. We tested four lipid extraction methods and chose an extraction based on quantitative measures of performance, *i.e.*, the number of variables, the total signal strength and the consistency of the method (see Results).

In order that data from large numbers of samples can be collected in one batch, both for testing extractions and for continued use in a pipeline, extractions must be carried out in parallel. Parallel extractions were carried out in this study using a 96-channel pipette mounted onto a movable platform (Integra Viaflo, ∼£15k). This allows preparation of 384w microplates for data collection.

Data collection poses a particular challenge in investigating whole systems as it requires large numbers of samples to be handled in parallel. High throughput techniques have emerged relatively recently in metabolomics, with several studies reporting thousands of samples per batch.^12–15^ For these analyses, extractions need to be automated^16^ with the minimum of steps to prepare samples.^17^ These and other methods have been reviewed^18–20^ and even tested.^11,21,22^ Liquid Chromatography Mass Spectrometry (LCMS) was chosen for this pipeline because it is the optimum approach to separate and measure the large number of lipids present in biological samples (only an order of magnitude less than that of proteins^41^). Recent advances in autosampler hardware mean that 384w microplates can now be used in commercially-available LCMS set-ups.

### 4.3. Data analysis

The typical approach to analysing big data at present is to use statistical tests and visualisations such as a principal component analysis (PCA). Fig. 2A is a PCA of a variety of sample types from different phylla, including plants, algae, fish, mammals and insects (Table S1, ESI†). Principal component or other ordinal analyses can be used to identify both sub-groups of samples and the variables drive the difference between two or more groups. However, this and other current methods can be limited for systemic analysis. Fig. 2B shows a PCA for the dissected tissues from queen bumble bees fed one of two different diets and Fig. 2C shows the dissected tissues from three species of fish. It is difficult to see how diet or taxonomy drive differences in the lipid metabolism of the two systems from ordinal analyses. The same problem is visible more acutely when lipidomics data from bees from two colonies fed different pollens are plotted (Fig. 2D), as the different parts of the system and the relationship between them are not clear. Our solution to this problem is to use a method for analysing the data that exploits the known connectivity between the different samples, such as the passing of nutrients between tissues within an organism or between trophic levels in an ecosystem.

Previously, we developed the concept of molecular traffic analysis and built software in R. Lipid traffic analysis (LTA) v1.0 and 2.3 were focused on spatial analyses within individuals.^8,9,33,42^ In order to be able to do systemic or network analysis suitable for colonies and ecosystems as well as individuals, we built LTA v3.0 in Python (https://pypi.org/project/lipidta/). This has additional features that are useful for complex networks (*vide infra*). The principle of traffic analysis in the context of metabolomics is based on the principle of lipid types. ***A***-type variables are lipids found in all compartments (tissues/sample types) of a given phenotype group. ***B***-type lipids are variables found in pairs of adjacent compartments, for example in the liver and the serum in mammals or the brain and ocular cortex in bees. ***U***-type variables are found only in one compartment for a given group. We introduce ***N***_**2**_-type variables that are for variables found in pairs of non-adjacent groups. The ***N***_**2**_-type is useful for identifying variables that exist independently or imply the existence of unexpected connections in a network, something that is useful in complex networks or networks that have not been fully explored. These lipid types are represented on a traffic analysis diagram alongside statistics to inform interpretation of the numbers. Jaccard–Tanimoto coefficients (JTCs, *J*) are used to show the overlap between the identities of the variables and associated *p* values were used as a non-parametric measure of the probability that the dissimilarity occurred by random chance (they are not the same as the *p* values used in *t*-tests).

We mapped the connectivity of samples in the proof-of-principle tests using their known metabolic connections (see Results). How these metabolites are distributed through two different systems shows how the two differ and thus the way they are controlled differs. This is the principal information output of a traffic analysis. We ran two proof-of-principle experiments, one was to understand how the control of biological systems differed between species (fish, Fig. 3) and colonies of *Bombus terrestris* fed different diets (Fig. 4).

### 4.4. Experimental information

#### 4.4.1 Materials, animals, consumables and chemicals

Solvents and fine chemicals were purchased from SigmaAldrich (Gillingham, Dorset, UK) and not purified further. Purified lipids were purchased from Avanti Polar lipids Inc. (Alabaster, Alabama, US). Plasticware was bought from Sarstedt (Darmstadt, Germany), ThermoFisher (Breda, NL), Fisher Scientific (Herfordshire, UK). Yeast strains were purchased from EUROSCARF (Oberursel, Germany). YPD medium was purchased from Formedium Ltd (Norfolk, UK). Human serum was purchased from SigmaAldrich (Gillingham, Dorset, UK). Mice were purchased from Harlan Laboratories Ltd (Alconbury, Cambridgeshire, UK) or Charles River Laboratories (UK). This research conformed to the Animals (Scientific Procedures) Act 1986 Amendment Regulations 2012 following ethical review by the University of Cambridge Animal Welfare and Ethical Review Body (AWERB). Unless otherwise indicated, mice were housed 3–5 per-cage in a temperature-controlled room (21 °C) with a 12 h light/dark cycle, with ‘lights on’ corresponding to 0600. The animals had *ad libitum* access to food and water. Standard chow diet was purchased from Safe diets (DS-105). Plant samples were purchased locally (Osterley Garden Centre, UK; PM Flowers, Kew, UK) or collected from the living collections at RBG Kew.

#### 4.4.2 Stock solutions

##### 4.4.2.1 GCTU

Guanidine (6 M guanidinium chloride) and thiourea (1.5 M) were dissolved in deionised H_2_O together and stored at room temperature out of direct sunlight.

##### 4.4.2.2 DMT

Dichloromethane (3 parts), methanol (1 part) and triethylammonium chloride (500 mg L^−1^) were mixed and stored at room temperature out of direct sunlight.

##### 4.4.2.3 Internal standards

The mixture of deuterated internal standards used in high throughput LCMS (Table S4, ESI†)

##### 4.4.2.4 XMI-AF

A mixture of xylene, methanol and isopropanol, 1:2:4, doped with 0.1% ammonium formate. The ammonium formate was constructed from stock solutions of ammonia (33%, aq.) and formic acid (100%, *d* = 1.2 g cm^−3^).

#### 4.4.3 Maintenance of animals and algae

##### 4.4.3.1 Mus musculus

All mouse procedures were conducted in accordance with the UK Home Office Animal (Scientific Procedures) Act 1986 Amendment Regulations 2012 following ethical review by the Aston University or University of Cambridge Animal Welfare and Ethical Review Body (AWERB). Mice were housed in specific-pathogen-free facilities with 12 h light and 12 h dark cycles. All mice were studied under fed conditions and at 24 °C. C57BL/6 mice from which heart, liver and adipose tissues were taken were fed a chow diet and maintained at Aston University's biomedical research facility. C57BL/6J mice from which brain, heart, adipose, liver, stomach, spleen, lung, skin and small intestine were taken were fed a chow diet and maintained at the University of Cambridge's animal facility at the Cambridge Biomedical campus.

##### 4.4.3.2 Apis mellifera

Frames of capped female brood were removed from three queen-right colonies of *Apis mellifera*, from an outdoor apiary at the John Krebbs Field Station, University of Oxford in 2021. Brood frames were suspended in a ventilated box inside a climate chamber at 34 °C and 60% relative humidity. Newly emerged bees were brushed off the frame each day and collected.

##### 4.4.3.3 Bombus terrestris

Commercial bumble bees for the colony feeding experiment were purchased from Agralan (Swindon, Wilts., UK) and kept in colonies in a laboratory incubator at the Insectary at RBG Kew (2022), and held at 28 °C and 60% humidity, fed a diet of irradiated, honeybee-collected pollen of either *Fagopyrum esculentum* (Buckwheat) or *Helianthus annuus* (sunflower) origin (Betterbee, Greenwich, US) and sucrose water (1 : 1 *w/v*). Bees fed chestnut, poppy or a combination of these pollens were purchased from Agralan Growers (Wiltshire, UK) and reared in a laboratory incubator at the Wytham research station (Oxford, UK), being held at 22–27 °C and 35–40% humidity.

All algae were cultivated in glass photobioreactors in liquid media at continuous light to OD_750_ of 1.5, harvested by centrifugation, frozen at −80 °C and freeze-dried.

*Desmodesmus quadricauda* (Turpin) Brébisson (strain Greifswald/15), Culture Collection of Autotrophic Organisms Institute of Botany, Czechia. Starting cultures were inoculated into SS medium, and cultivated at 30 °C, 750 μmol photons m^−2^ s^−1^, 2% *v/v* CO_2_.^43^

*Chlamydomonas reinhardtii* wild type 21gr (CC-1690) Chlamydomonas Resource Center at the University of Minnesota, St. Paul, MN, USA. Starting cultures were inoculated into HS medium, and cultivated at 30 °C, 500 μmol photons m^−2^ s^−1^, 2% *v/v* CO_2_.^44^

*Galdieria sulphuraria* (Galdieri) Merola, 002, Algal Collection of the University “Federico II” of Naples, Italy. Starting cultures were inoculated into Galdieria medium, *p*H 3, and cultivated at 40 °C, 500 μmol photons m^−2^ s^−1^, 2% *v/v* CO_2_.^45^*Hibberdia magna* K-1175, Norwegian Culture Collection of Algae, Norway. Starting cultures were inoculated into WC medium, and cultivated at 20 °C, 150 μmol photons m^−2^ s^−1^, 1% *v/v* CO_2_.^46^

#### 4.4.4 Sample preparation

##### 4.4.4.1 Mammalian tissues

Tissues were prepared as previously described.^10^ Briefly, the relevant tissue/organ was stored at −80 °C and homogenised immediately in the presence of GCTU (see Table S1 for ratio, ESI†) using a hand-held homogeniser (Tissue Tearor, 14 mm head, <2 min) and the resulting homogenate stored (−80 °C) until lipid extraction. Samples from >10 mice were pooled to prepare the pooled stocks of adipose, heart, brain and liver. Individual mouse tissues used were from one individual that had been fed a chow diet. Human plasma and whole blood were used as supplied.

##### 4.4.4.2 Insect tissues

###### 4.4.4.2.1 Apis mellifera

Newly emerged bees (*Apis mellifera*) were collected and dissected before they ingested any external feed (<6 h). Bees were pinned to a cork mat, on ice, before prompt dissection of the brain, HPG, gut, eye and optical lobe, and fat body. The resulting tissues stored briefly on wet ice until completion of all animals’ dissection, whereupon all samples were stored at −80 °C until they were homogenised. Frozen samples were covered in GCTU (see Table S1 for ratio, ESI†) before being homogenised (Tissue Tearor, 4 mm head, low/medium power, 1–2 min). The resulting homogenates were stored (−80 °C) until lipid extraction. Aged samples were stored at −80 °C except for a period of one week where they were refrigerated (5 °C).

###### 4.4.4.2.2 Bombus terrestris

Queens from the *B. terrestris* colonies were collected from the colony as it was being dismantled, and dissected. Animals were culled (−20 °C) and pinned to a cork or neoprene mat before prompt dissection of the brain, ovaries, thoracic muscle, crop, mid-gut, hindgut, venom gland, eye and ocular cortex, and fat body. The resulting tissues stored briefly on wet ice until completion of all animals’ dissection, whereupon all samples were stored at −80 °C until they were homogenised. Frozen samples were covered in GCTU (see Table S1 for ratio, ESI†) before being homogenised (Tissue Tearor, 9 mm head, low/medium power, 1–2 min). The resulting homogenates were stored (−80 °C) until lipid extraction.

##### 4.4.4.3 Fish

Fresh, whole, healthy, individual examples of fish were used. *Dicentrarchus labrax* and *Sparus aurata* were acquired from Mediterranean farm waters. *Scomber scombrus* were Atlantic wild-caught off the cost of Spain. All fish were landed at Grimsby. *Salmo salar* were farmed in Scotland in Loch Duart. All fish were transported to the dissection centre (Cambridge) at −80 °C. For dissection, the fish were thawed to 2 °C and dissected rapidly in a refrigerated room (2 °C) and the tissues and whole blood frozen at −20 °C before being frozen and stored at −80 °C.

##### 4.4.4.4 Whole yeast (Saccharomyces cerevisiae)

The diploid homozygous deletion strain *erg*3Δ/*erg*3Δ (EUROSCARF accession number Y32667) and the isogenic control strain BY4743 were cultured (1 L, 30 °C, YPD medium, orbital shaking) for three days to reach the stationary phase. The cultures were centrifuged (720*g*, 5 min) and the medium discarded. The pelleted yeast cells were transferred to a Falcon tube (50 mL) and resuspended in GCTU (5 mL) before being flash-frozen (liquid nitrogen), freeze-dried and stored (−80 °C, 24 months). The solid was dispersed in water (double-distilled, 10 mL), frozen (−80 °C) and freeze-dried again.

##### 4.4.4.5 Plant tissues

Various tissues from a phylogenetically varied set of four terrestrial plants was used (Table S1, ESI†). Sap was collected from stems by application of pressure (hand) on obliquely-cut sections of stem. Resulting liquid was diluted (GCTU, 50 μL) and stored (−80 °C) until extraction. Leaves, petals and mature capsules were sliced or diced using a razor blade to give fibres that were typically <5 mm long, before being covered in water (ddH_2_O, 5–10 mL), frozen (−80 °C for storage, then −196 °C) and freeze-dried. The freeze-dried samples were all covered in GCTU (typically 10× *v/v*, see Table S1, ESI†) left to stand (2–6 h) and then homogenised (14 mm head, full power, 1–2 min). The homogenates were stored (−20 °C) before being used. Pollen samples were dispersed in GCTU (25 : 1 *v/w*).

#### 4.4.5 Preparation of tissues for high throughput extraction of the lipidome

##### 4.4.5.1 Quality control samples

QC samples were used to assess whether signal strength correlated with concentration. Thus a range of sample types was combined randomly into two QC stocks. Tissues homogenised in GCTU from *Mus musculus* (brain, adipose, liver), Bees (whole, adult, pupa and larva, wax, frass), plant (mixed pollen, leaf, algae) were combined. These were pipetted onto the plate at 25, 50 or 100% (7.5, 15 or 30 μL). Three technical replicated of each concentration were pipetted onto each 96w plate. Each QC stock was used at least once on each 384w plate, with both run on all 96w plates where possible.

##### 4.4.5.2 High throughput extraction of the lipidome

Extractions were carried out as closely as possible to the original instructions for each method (BAD,^24^ DMT,^10^ TBM^16^), with adjustments being made only for high throughput sample handling. Before use on lipid experiments, the autosampler and chromatography system were tested using a stock of polar metabolites (proline, leucine, theobromine and catechin). Testing showed the CV of all four of these metabolites was <3%, and that of catechin 1.1% (96 samples). This indicated that the hardware was remarkably consistent and thus well placed for larger-scale data acquisition of more difficult metabolites such as lipids.

##### 4.4.5.3 BAD

Liquid homogenates of tissue preparations were injected into the appropriate well of a 96-well extraction plate (glass-coated, SureSTART™ WebSeal™, 2.0 mL per well; volumes of homogenate shown in Table S1, ESI†) along with appropriate blanks and QCs, followed by internal standards (mixture of internal standards in methanol/xylene/isopropanol, 150 μL, see Table S4, ESI†), water (500 μL), and chloroform (500 μL), using a 96-channel pipette (VIAFLO 96/384, Integra Biosciences, Berkshire, UK). The mixture was agitated (96-channel pipette) before being centrifuged (3.2k × *g*, 2 min). A portion of the organic solution (20 μL) was transferred to a high-throughput plate (384-well, glass-coated, SureSTART™ WebSeal™ Plate+) before being dried (N_2(g)_).

##### 4.4.5.4 DMT

Liquid homogenates of tissue preparations were injected into the appropriate well of a 96-well extraction plate (glass-coated, SureSTART™ WebSeal™, 2.0 mL per well; volumes of homogenate shown in Table S1, ESI†) along with appropriate blanks and QCs, followed by internal standards (mixture of internal standards in methanol/xylene/isopropanol, 150 μL, see Table S4, ESI†), water (500 μL) and DMT (500 μL) using a 96-channel pipette (VIAFLO 96/384, Integra Biosciences, Berkshire, UK) and GripTips (300 μL, Green choice). The mixture was agitated thoroughly (96-channel pipette) before being centrifuged (3.2k × *g*, 2 min). A portion of the organic solution (20 μL) was transferred to a high-throughput plate (384-well, glass-coated, SureSTART™ WebSeal™ Plate+) before being dried (N_2(g)_).

##### 4.4.5.5 TBM

Liquid homogenates of tissue preparations were injected into the appropriate well of a 96-well extraction plate (glass-coated, SureSTART™ WebSeal™, 2.0 mL per well; volumes of homogenate shown in Table S1, ESI†) along with appropriate blanks and QCs, followed by internal standards (mixture of internal standards in methanol/xylene/isopropanol, 150 μL, see Table S4, ESI†), water (500 μL) and TBME (500 μL). The mixture was centrifuged (3.2k × *g*, 2 min). A portion of the organic solution (20 μL) was transferred to a high throughput plate (384-well, glass-coated, SureSTART™ WebSeal™ Plate+) before being dried (N_2(g)_).

##### 4.4.5.6 EAT

This procedure is novel to the present study, using ethyl acetate saturated with triethylammonium chloride (<500 mg L^−1^), referred to as EAT. Liquid homogenates of tissue preparations were injected into the appropriate well of a 96-well extraction plate (glass-coated, SureSTART™ WebSeal™, 2.0 mL per well; volumes of homogenate shown in Table S1, ESI†) along with appropriate blanks and QCs, followed by internal standards (mixture of internal standards in methanol/xylene/isopropanol, 150 μL, see Table S4, ESI†), water (500 μL) and EAT (500 μL) using a 96-channel pipette (VIAFLO 96/384, Integra Biosciences, Berkshire, UK). The mixture was agitated thoroughly (96-channel pipette) before being centrifuged (3.2k × *g*, 2 min). A portion of the organic solution (20 μL) was transferred to a high-throughput plate (384-well, glass-coated, SureSTART™ WebSeal™ Plate+) before being dried (N_2(g)_).

Once extracts from all four of the 96-well plates had been placed in the 384 well plate (glass-coated, SureSTART™ WebSeal™ Plate+), the dried films were re-dissolved (XMI-AF, 80 μL per well) and the plate was heat-sealed with aluminium foil (AB-0757, Fisher Scientific) and queued immediately, with the first injection within 5 min. The extractions were timed so that the instrument was available immediately after the completion of extractions.

##### 4.4.5.7 Liquid chromatography mass spectrometry

All LCMS was carried out using a Thermo Scientific Vanquish LC system with a quaternary pump, equipped with a Thermo Scientific Hypersil GOLD LCMS C_18_ column (50 × 2.1 mm, particle size 1.9 μm) and a Thermo Scientific Orbitrap Fusion® MS with an H-ESI ioniser. Eluents were acetonitrile (LCMS grade); water (deionised, ammonium formate 0.1% *v/v* added fresh, prepared from ammonia and formic acid and pipetted by volume); isopropanol (LCMS grade). The chromatographic method is shown in Table 1. Once collected, the *.raw data files were stored, backed up and data processing begun. Mass spectrometric data were collected in positive ionisation mode at a resolution of 120 000 (*m*/*z* 200) with the H-ESI spray voltage set to 2.86 kV, nitrogen gas flows of 45 (sheath), 5 (auxiliary) and 1 (sweep) arbitrary units, and ion transfer tube and vaporizer temperatures of 300 °C and 350 °C. The AGC was set to Standard (Full Scan 1 000 000 and SIM/PRM 200 000) with a maximum ion injection time of 200 ms. The mass acquisition window was *m*/*z* 480–1100, with the fluoranthene cation (*m*/*z* 202.077) used for internal mass calibration.

**Table 1:** Chromatographic method for analytical separation of lipids and triglycerides for high throughput lipidomics

Chromatographic method for phospholipid/triglyceride extracts			
Time (min)	Acetonitrile	Water^a^	Isopropanol
0	15	40	45
2	15	32.5	52.5
2.1	15	25	60
6	15	20	65
12	15	17	68
12.1	15	40	45
15	15	40	45

a Ammonium formate (0.1%) was added fresh to water shortly before use.

##### 4.4.5.8 Data processing (unmatched IDs)

All LCMS *.raw files generated were converted into *.mzXML files using Proteowizard(Chambers) (3.0.23). Converted data files were processed using the CAMERA package using R (v3.6.0), with peak picking performed using a “centwave” method that allows for the deconvolution of closely eluting or slightly overlapping signals.^26^ Metabolite features were then defined as any peak with an average intensity at least 5 times higher in analytical samples relative to the abundance seen in the extraction blanks. All signals that passed were present in ≥90% of samples in at least 1 sample type.

##### 4.4.5.9 Data processing (matched IDs)

AnalyzerPro® XD (SpectralWorks, Ltd) was used for processing data. A Target library (*.swix) was constructed from a generated *m*/*z* and lipid ID list, with known samples and Internal Standards used to determine retention times (*R*_t_). All LCMS data *.raw files were uploaded to the software and processed (Mass range 400–1200 Da; *R*_t_ window 0.5–18.5 min; area threshold 100k; detection width 0.25 min; Mass accuracy 3 d.p.). The signals (matched and unmatched) were recorded in a CSV file that was subsequently used for quality checks. Variables with an average signal strength >3× that of the same signal/*R*_t_ in the blank samples were regarded as passing the *S/N* test. QC samples were used to assess whether the signal strength correlated with the concentration, *i.e.* the correlation between 0.25×, 0.5× and 1.0× QCs against 25, 50 and 100% was calculated separately for the two QC stocks. QC stock 1 consisted of mixtures of freeze-dried leaf, pollen and whole bees, whereas QC stock 2 consisted of brain heart and liver homogenates from *Mus musculus*, and belly, skin, heart and liver from *Dicentrarchus labrax*.

All signals for which the correlation was found to be >0.75 for at least one of the QC stocks used was regarded as passing the QC test. 3198 variables passed both tests, across all samples.

##### 4.4.5.10 Traffic analysis

Traffic analyses were carried out using v3.0 of the LTA software, updated from v2.3^7,9,33^ for this study and is available as open source software from GitHub (https://pypi.org/project/lipidta/). The analyses in this study was based on known maps of the metabolic systems studied. Statistics are provided to aid interpretation of traffic analysis diagrams. Jaccard–Tanimoto coefficients (JTCs, *J*) and associated *p* values were used as a non-parametric measure of the distinctions between lipid variables associated with phenotype(s). These were used to calculate the overlap between the identities of the variables and the probability that this occurred by random chance, respectively. Variables were regarded as present in a given group if they had a signal strength >0 in ≥66% of samples that group.

##### 4.4.5.11 Software

Microsoft Office 365 Excel was used for handling spreadsheets, data processing and signal sheet preparation and storage (*.xlsx format). Figures were drawn in Powerpoint or Origin 2018. LCMS data were proceed using R (v3.6.0) or AnalyzerPro® XD (SpectralWorks Ltd).

## Author contributions

SF conceived the project, collected and analysed data and wrote the manuscript. SF, CM, SGS and PCS designed experiments. DFT, DW, AJW, SV, DS and JS did all animal husbandry and dissections. SF, CM, DW, DS, DFT, JS, MV, EB, JC, SGS, TAKP, AJW and SV produced or collected samples, carried out experiments and optimisations. SF, AK, RPC and DC devised advances in LTA software, with RPC and DC writing all code and testing with SF. RPC wrote all Python code from the original R code by DC. SF and GCK developed instrumentation and methods. JM, SGS and SF processed data. PCS, GAW, AK, SV, SEO and AVP wrote the original grant proposals. SF, SGS and PCS supervised the project and revised the manuscript with comments from all authors. All authors commented on the manuscript and approved the final version.

## Data availability

The raw data, as *.raw files, for all the samples run in this study are available from The Knowledge Network for Biocomplexity (https://knb.ecoinformatics.org/view/doi:10.5063/F15B00XJ), with the DOI 10.5063/F15B00XJ. The processed mass spectrometry data can be found in the SI and from the communicating authors. The code for LTA v3.0 is publicly available through https://pypi.org/project/lipidta/.

## Conflicts of interest

The authors have no competing interests to declare.

## Supplementary Material

MO-020-D4MO00083H-s001

MO-020-D4MO00083H-s002

MO-020-D4MO00083H-s003

MO-020-D4MO00083H-s004
