# Supplementary material for: Systemic analysis of lipid metabolism from individuals to multi-organism systems
Source: Mol Omics. 2024 Sep 9;20(9):570–83. doi: 10.1039/d4mo00083h (PMC11381968; doi:10.1039/d4mo00083h)
Supplement: MO-020-D4MO00083H-s002 [file MO-020-D4MO00083H-s002.pdf]

Supplementary Information (SI) for Molecular Omics.
This journal is © The Royal Society of Chemistry 2024

Supplementary Figures

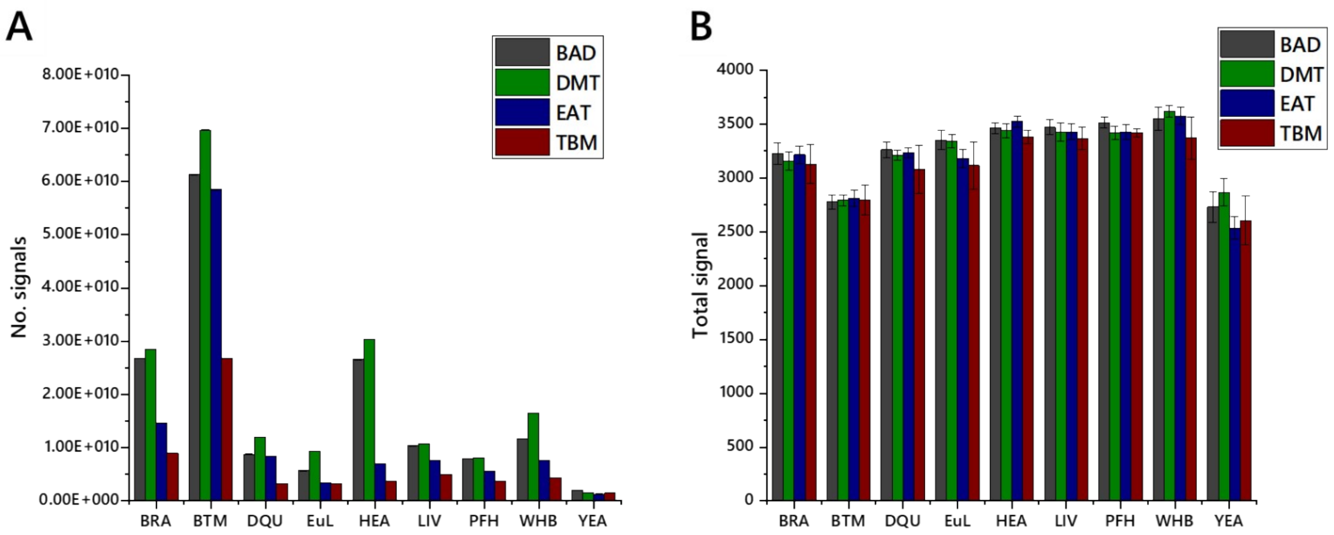

Fig. S1. The performance of four lipid extraction methods on nine sample types. Panel **A**, the total number of variables of unmatched *m/z* signals found for four extractions across nine sample types, that passed background and QC checks. Panel **B**, the total signal of all unmatched *m/z* signals found for four extractions across nine sample types, that passed background and QC checks. Samples were drawn from stock materials (see methods). BAD, Bligh & Dyer extraction applied to high throughput extraction[1]; DMT, dichloromethane-methanol-triethylammonium chloride[2]; EAT, ethyl acetate with triethylammonium chloride; TBM, *tert*-butylmethylether extraction, as described by Matyash *et al.*[3]. BRA, pooled brains from *Mus musculus*; BTM, milk from *Bos taurus*; DQU, whole pooled *Desmodesmus quadricauda*; EuL, leaves from *Eucalyptus perriniana*; HEA, pooled hearts from *Mus musculus*; LIV, pooled livers from *Mus musculus*; PFH, polyfloral pollen; WHB, whole *Bombus terrestris*, pooled; YEA, *Saccharomyces cerevisiae* BY 4743. Error bars represent standard deviation based on 10 mesaurements.

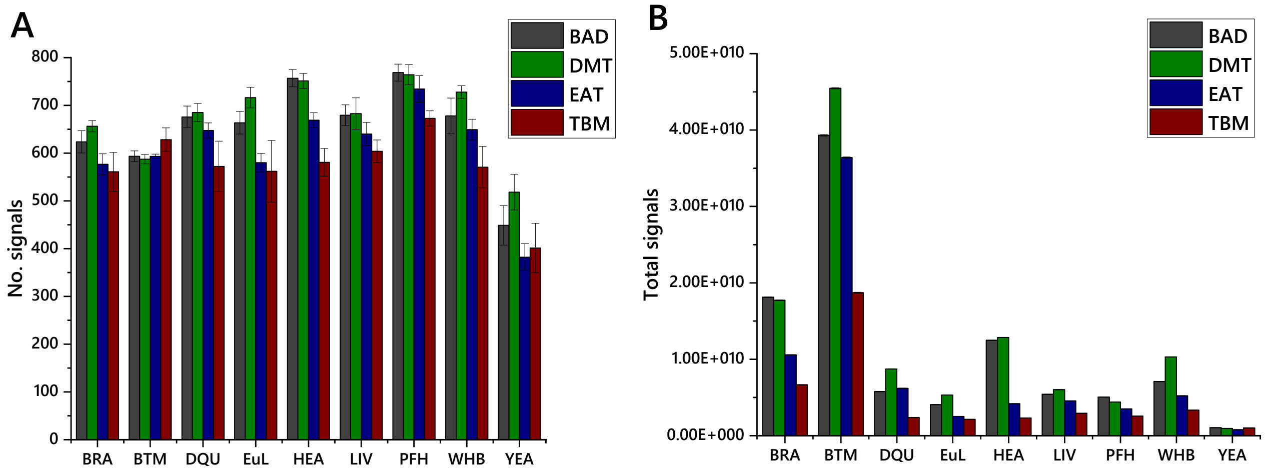

Fig. S2. The performance of four lipid extraction methods on nine sample types, processed using a target library. Panel **A**, the total number of variables of matched *m/z* signals found for four extractions across nine sample types, that passed background and QC checks. Panel **B**¸ the total signal of all matched *m/z* signals found for four extractions across nine sample types, that passed background and QC checks. Samples were drawn from stock materials (see methods). BAD, Bligh & Dyer extraction (high throughput extraction)[1]; DMT, dichloromethane-methanol-triethylammonium chloride[2]; EAT, ethyl acetate with triethylammonium chloride; TBM, *tert*-butylmethylether extraction, as described by Matyash *et al.*[3]. BRA, pooled brains from *Mus musculus*; BTM, milk from *Bos taurus*; DQU, whole pooled *Desmodesmus quadricauda*; EuL, leaves from *Eucalyptus perriniana*; HEA, pooled hearts from *Mus musculus*; LIV, pooled livers from *Mus musculus*; PFH, polyfloral pollen; WHB, whole *Bombus terrestris*, pooled; YEA, *Saccharomyces cerevisiae* BY 4743.

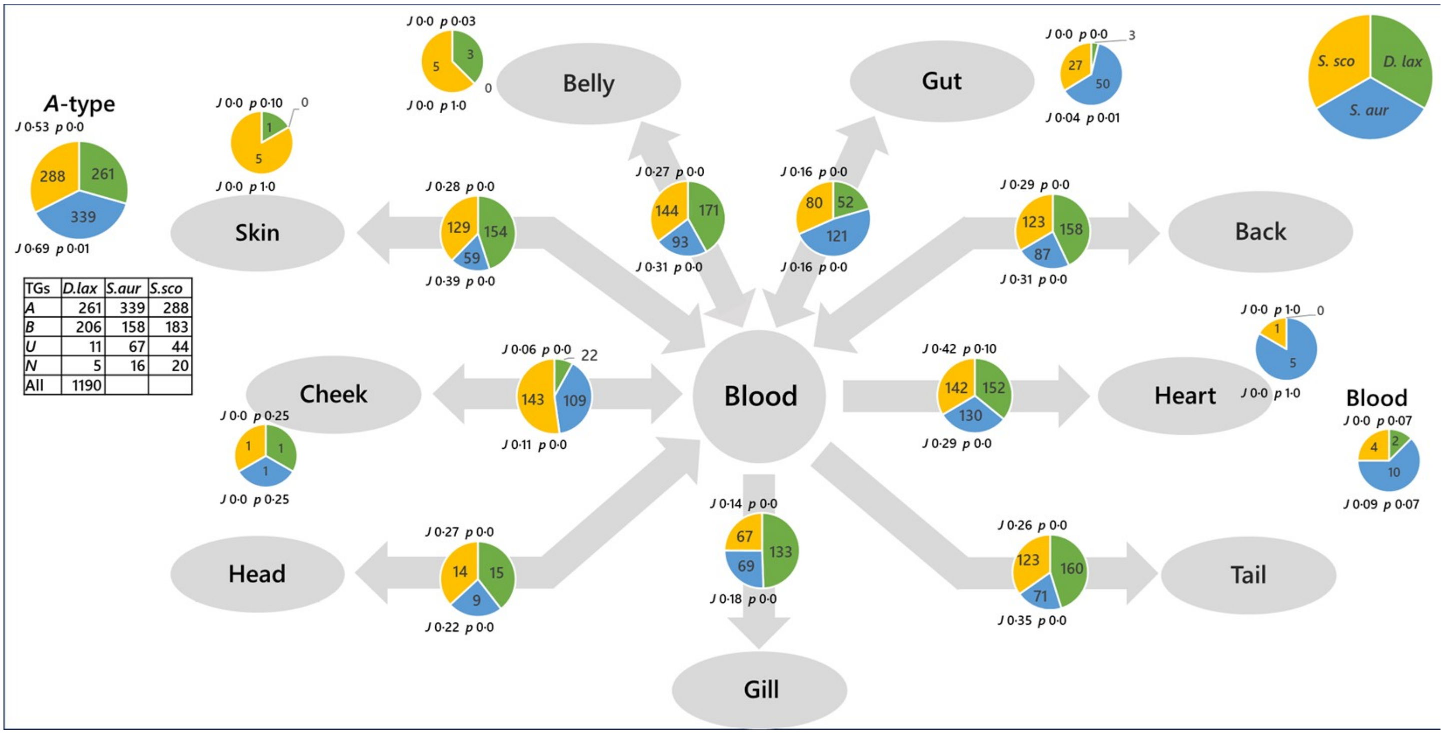

Fig. S3. A switch analysis of triglycerides in *Dicentrarchus labrax* (seabass) against *Sparus aurata* (bream) and *Scomber scombrus* (mackerel). The pie chart in the top left shows the number of ubiquitous lipid variables for that network, for each phenotype (***A***-type variables). Pie charts on arrows represent variables found in the two adjacent compartments (***B***-type variables). Smaller pie charts represent isolated variables (***U***-type). ***J*** represents the Jaccard-Tanimoto coefficient for the comparison, with accompanying ***p*** value, as a measure of the similarity between the variables identified in the two phenotypes for each comparison. The ***p*** value shown represents the probability that the difference between the lists of variables for the two phenotypes occurred by random chance. TGs include all adducts of whole TGs and the DGs arising from in-source fragmentation of TGs during data collection.

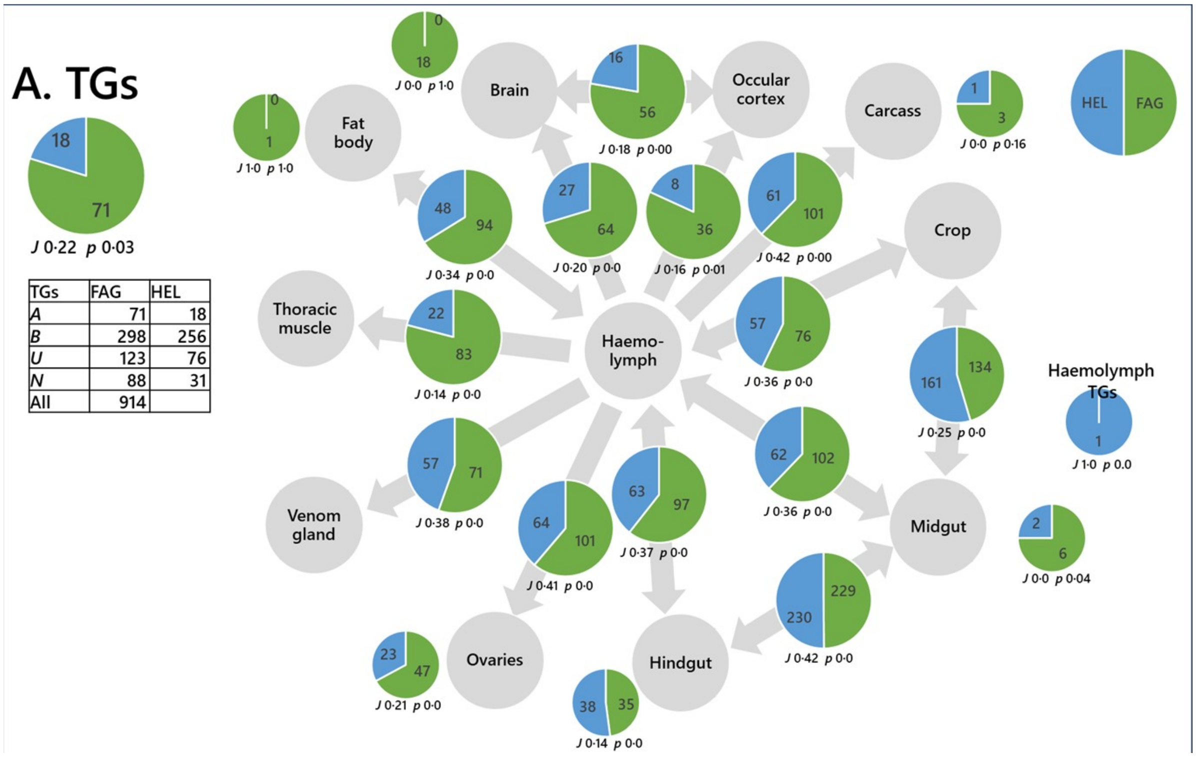

TGs	FAG	HEL
A	71	18
B	298	256
U	123	76
N	88	31
All	914	

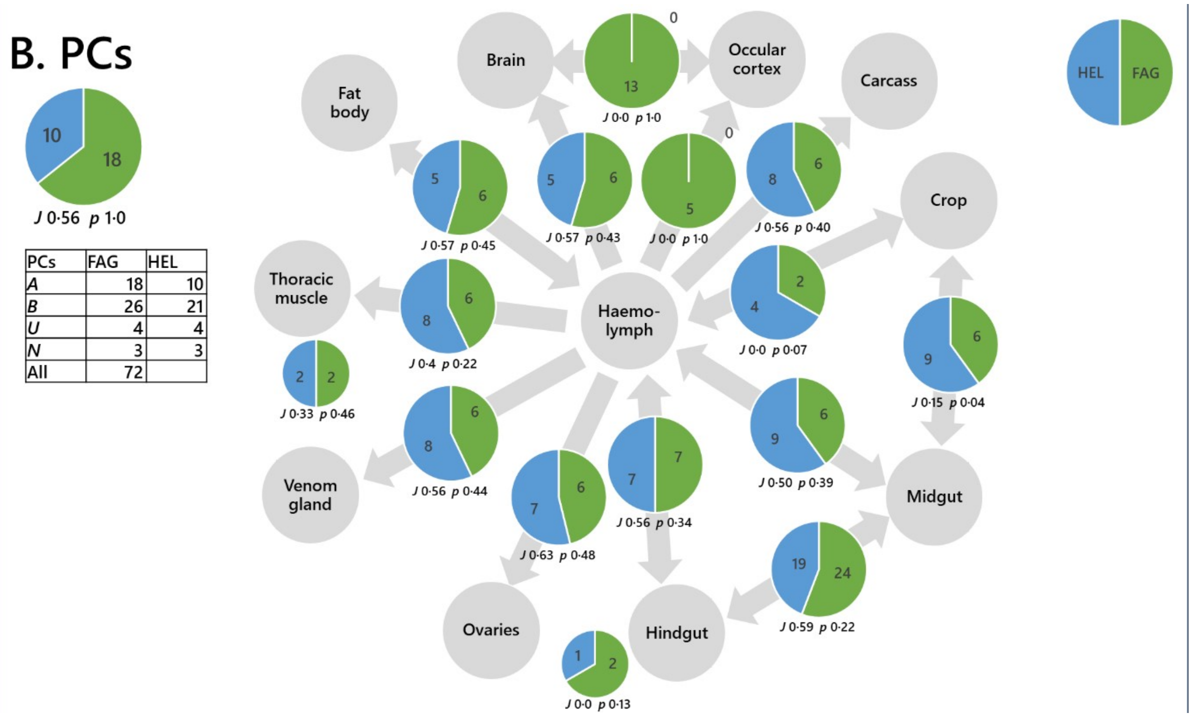

PCs	FAG	HEL
A	18	10
B	26	21
U	4	4
N	3	3
All	72	

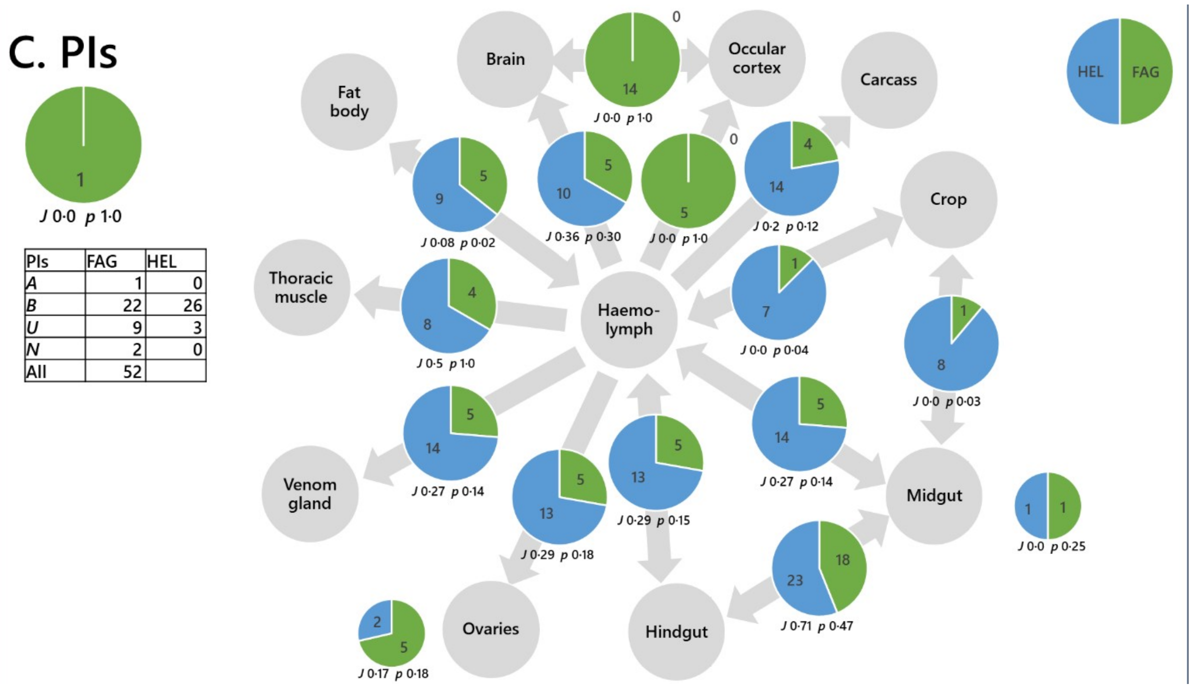

PIs	FAG	HEL
A	1	0
B	22	26
U	9	3
N	2	0
All	52	

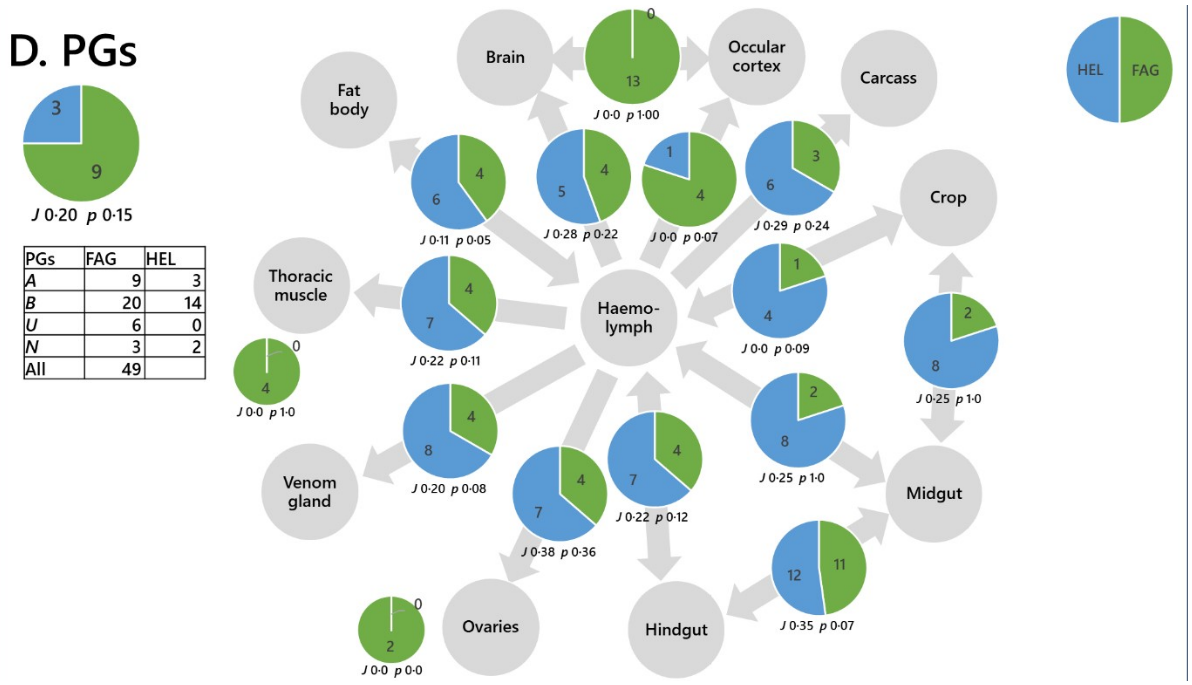

PGs	FAG	HEL
A	9	3
B	20	14
U	6	0
N	3	2
All	49	

Fig. S4. Switch analyses of phospholipid and triglyceride variables in Queen *Bombus terrestris* bees fed either *Fagopyrum tataricum* (FAG) or *Helianthus annuus* (HEL) pollen. Panel **A**, Switch Analysis of triglycerides; **B**, Switch Analysis of phosphatidylcholines; **C**, Switch Analysis of phosphatidylinositols; **D**, Switch Analysis of phosphatidylglycerols. The pie chart in the top right shows the number of ubiquitous lipid variables for that network, for each phenotype (***A***-type variables). Larger pie charts (on the arrows) represent variables found in the two adjacent compartments (***B***-type variables). Smaller pie charts represent isolated variables (***U***-type). ***J*** represents the Jaccard-Tanimoto coefficient for the comparison, with accompanying ***p*** value, as a measure of the similarity between the variables identified in the two phenotypes for each comparison. The ***p*** value shown represents the probability that the difference between the lists of variables for the two phenotypes occurred by random chance.

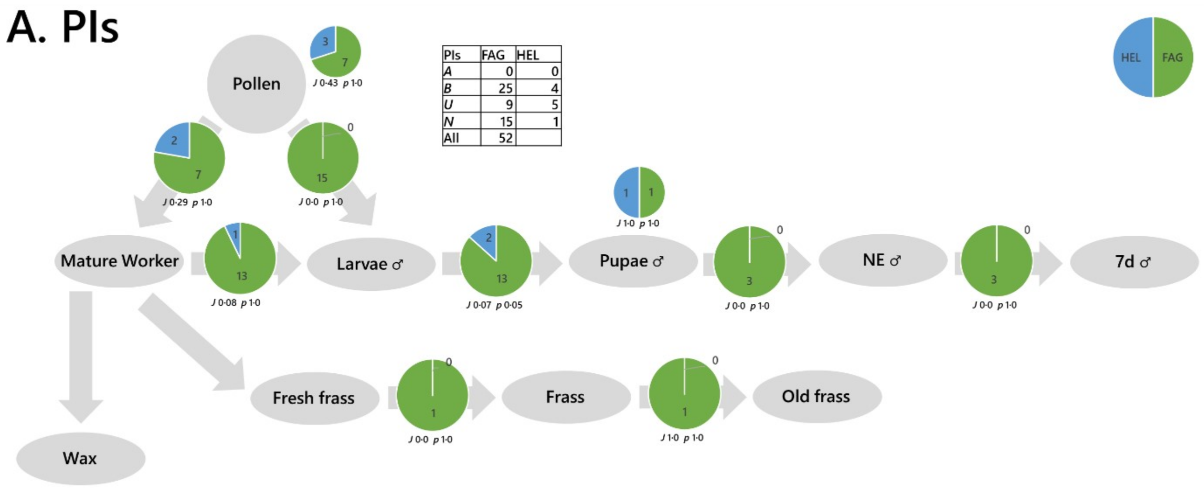

PIs	FAG	HEL
A	0	0
B	25	4
U	9	5
N	15	1
All	52	

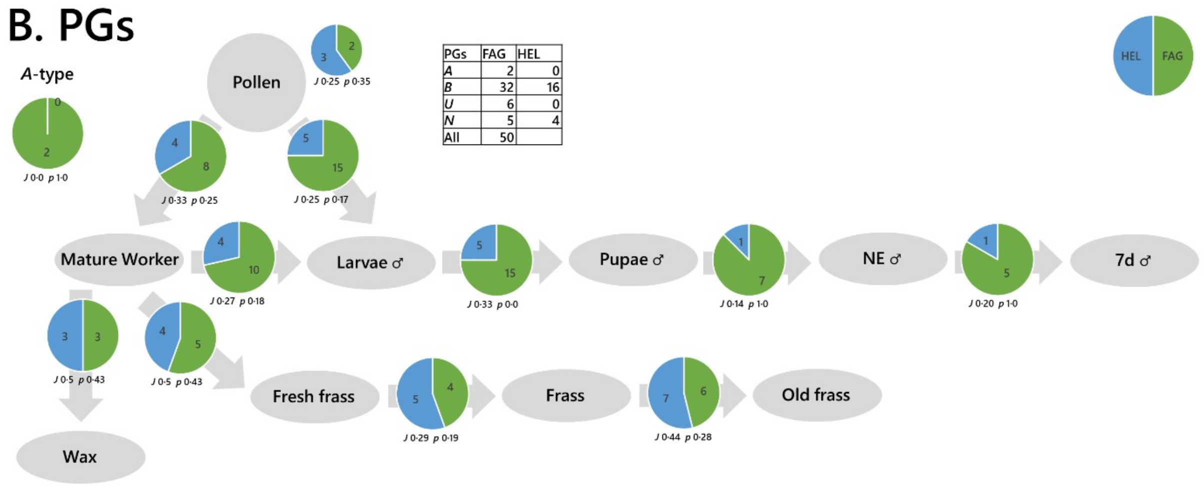

PGs	FAG	HEL
A	2	0
B	32	16
U	6	0
N	5	4
All	50	

Fig. S5. Switch analyses of phospholipid and triglyceride variables in *Bombus terrestris* colonies fed either *Fagopyrum tataricum* (FAG) or *Helianthus annuus* (HEL) pollen. Panel **A**, Phosphatidylinositols; **B**, Phosphatidylglycerols. The pie chart in the top right shows the number of ubiquitous lipid variables for that network, for each phenotype (***A***-type variables). Larger pie charts (on the arrows) represent variables found in the two adjacent compartments (***B***-type variables). Smaller pie charts represent isolated variables (***U***-type). ***J*** represents the Jaccard-Tanimoto coefficient for the comparison, with accompanying ***p*** value, as a measure of the similarity between the variables identified in the two phenotypes for each comparison. The ***p*** value shown represents the probability that the difference between the lists of variables for the two phenotypes occurred by random chance.

TABLES

<<see excel spreadsheet, attached>>

Table S1. Sample list and preparation of tissues used in the present study. The purpose of the ratio is to give a chemically and biologically stable, pipettable solution in which 1-5 µg of lipid can be transferred in 5-60 µL liquid. [1]Ratio of GCTU to fresh weight (=1). This is provided as a guide, tissues with more/less fatty material may need different ratios of buffer to sample; [2]Material added to 1 mL of GCTU dispersion; [3]samples were freeze-dried before mechanical disruption/dispersion with a hand-held homogeniser, see instructions; [4]Samples stored at 5°C for a week before homogenisation. Pooled stocks used in the present study represent homogenates from at least 10 individuals. Pollen not marked as fresh was collected by bees.

CV	*D. quad.* (whole)	*Eucalyptus per.* (leaf)	Polyfloral pollen	*Bombus terrestris* (whole)	*Saccharomyces cerevisiae* (whole)	*Mus musculus* (brain)	*Mus musculus* (heart)	*Mus musculus* (liver)	*Bos taurus* (milk)	Sum
**30%**										
**BAD**	328	306	753	493	270	509	603	594	242	4098
**DMT**	293	239	653	450	341	535	570	381	349	3811
**EAT**	383	437	751	581	279	616	757	449	374	4627
**TBM**	70	152	811	257	193	288	319	480	177	2747
**20%**										
**BAD**	69	109	278	146	85	162	206	208	88	1351
**DMT**	54	51	314	167	111	236	227	87	136	1383
**EAT**	120	190	341	205	89	250	228	92	154	1669
**TBM**	9	36	398	48	46	57	61	131	33	819
**15%**										
**BAD**	12	41	121	44	25	48	67	69	32	459
**DMT**	8	13	172	71	33	108	96	18	41	560
**EAT**	32	76	184	97	33	120	60	19	63	684
**TBM**	1	8	225	23	25	25	15	36	8	366

Table S2. The number of variables with a coefficient of variation below three thresholds. Signals are unmatched *m/z* signals of isolates of four extractions across nine sample types that passed background and QC checks. Samples drawn from stock materials (see methods). BAD, Bligh & Dyer extraction applied to high throughput extraction[1]; DMT, dichloromethane-methanol-triethylammonium chloride[2]; EAT, ethyl acetate with triethylammonium chloride; TBM, *tert*-butylmethylether extraction[3].

CV	*D. quad.* (whole)	*Eucalyptus per.* (leaf)	Polyfloral pollen	*Bombus terrestris* (whole)	*Saccharomyces cerevisiae* (whole)	*Mus musculus* (brain)	*Mus musculus* (heart)	*Mus musculus* (liver)	*Bos taurus* (milk)	Sum
**30%**										
**BAD**	90	82	90	178	153	125	144	142	80	1084
**DMT**	96	78	105	193	126	133	91	172	120	1114
**EAT**	114	107	89	193	144	132	117	157	123	1176
**TBM**	20	31	74	185	108	74	103	67	44	706
**20%**										
**BAD**	51	56	63	117	104	93	98	107	58	747
**DMT**	57	44	73	138	88	103	46	120	88	757
**EAT**	87	78	67	155	116	103	72	118	88	884
**TBM**	9	17	58	142	54	49	75	34	33	471
**15%**										
**BAD**	2	17	16	35	25	23	35	28	22	203
**DMT**	5	4	24	40	37	40	6	31	30	217
**EAT**	25	22	5	54	30	33	57	14	23	263
**TBM**	1	5	26	56	4	5	22	8	3	130

Table S3. The number of variables with a coefficient of variation below three thresholds. Signals are Lipid-ID matched *m/z* signals of isolates of four extractions across nine sample types that passed background and QC checks. Samples drawn from stock materials (see methods). BAD, Bligh & Dyer extraction applied to high throughput extraction[1]; DMT, dichloromethane-methanol-triethylammonium chloride[2]; EAT, ethyl acetate with triethylammonium chloride; TBM, *tert*-butylmethylether extraction[3].

	Lipid	Expected mass (mg)	Concentration (nM)	*m/z* (+ve ionis. Mode, +H+)	*m/z* (+ve ionis. Mode, +NH4+)	*m/z* (+ve ionis. Mode, +Na+)
1	LPC	1	1.889	529.3989	-	551.3811
2	SM	1	1.361	**734.7684**	-	756.7506
3	PE	10	13.356	748.7241	-	770.7067
4	PS	10	12.615	792.7140	-	814.6965
5	PI	1	1.204	830.5767	847.6030	**852.5583**
6	PC	10	11.641	859.06	-	881.0383
7	TG(light)	1	1.232	-	771.7224	776.6774
8	TG(heavy)	1	1.327	-	829.7979	834.7527
9	DGDG*	10	13.268	949.6827	966.7093	971.6647
10	MGDG*	10	13.268	759.5986	776.6252	781.5806

Table S4. The Internal Standards used. Standards were labelled with at least 6 deuterium atoms and used without purification. *Not deuterated.
